# Handgrip strength is correlated with activities of daily living, balance, and body composition in patients with thoracolumbar compression fracture

**DOI:** 10.1097/MD.0000000000033141

**Published:** 2023-03-03

**Authors:** Hirokazu Inoue, Yukinori Hayashi, Hideaki Watanabe, Hideaki Sawamura, Yasuyuki Shiraishi, Ryo Sugawara, Atsushi Kimura, Masaaki Masubuchi, Katsushi Takeshita

**Affiliations:** a Department of Orthopaedics, Jichi Medical University, Shimotsuke, Japan; b Department of Orthopaedic Surgery, Shiobara Spring Hospital, Nasushiobara, Japan; c Department of Pediatric Orthopaedic Surgery, Jichi Children’s Medical Center, Shimotsuke, Japan.

**Keywords:** activities of daily living, bioelectrical impedance analysis, handgrip strength, phase angle, sarcopenia, vertebral compression fracture

## Abstract

This study assessed the relationship between handgrip strength (HGS) and activities of daily living, balance, walking speed, calf circumference, body muscle, and body composition in elderly patients with thoracolumbar vertebral compression fracture (VCF). A cross-sectional study in a single hospital was performed with elderly patients diagnosed with VCF. After admission, we evaluated HGS, 10-meter walk test (speed), Barthel Index, Berg Balance Scale (BBS), numerical rating scale of body pain, and calf circumference. We examined skeletal muscle mass, skeletal muscle mass index, total body water (TBW), intracellular water, extracellular water (ECW), and phase angle (PhA) in patients with VCF using multi-frequency direct segmental bioelectrical impedance analysis after admission. A total of 112 patients admitted for VCF were enrolled (26 males, 86 females; mean age 83.3 years). The prevalence of sarcopenia according to the 2019 Asian Working Group for Sarcopenia guideline was 61.6%. HGS was significantly correlated with walking speed (*P* < .001, *R* = 0.485), Barthel Index (*P* < .001, *R* = 0.430), BBS (*P* < .001, *R* = 0.511), calf circumference (*P* < .001, *R* = 0.491), skeletal muscle mass index (*P* < .001, *R* = 0.629), ECW/TBW (*P* < .001, *r* = −0.498), and PhA (*P* < .001, *R* = 0.550). HGS was more strongly correlated with walking speed, Barthel Index, BBS, ECW/TBW ratio, and PhA in men than women. In patients with thoracolumbar VCF, HGS is associated with walking speed, muscle mass, activities of daily living measured using the Barthel Index, and balance measured using BBS. The findings suggest that HGS is an important indicator of activities of daily living, balance, and whole-body muscle strength. Furthermore, HGS is related to PhA and ECW/TBW.

## 1. Introduction

The prevalence of osteoporosis is increasing in line with the aging worldwide population, leading to a greater prevalence of vertebral compression fractures (VCFs) and hip fractures caused by falls. Population studies have indicated that 8% of adults over 50 years of age have osteoporosis of the lumbar spine, and the prevalence of VCF adjusted for age and sex is estimated at 117 per 100,000 person-years.^[1]^ VCFs are more common in elderly populations, causing gait disturbances and decreased activities of daily living (ADL). The Asian Working Group for Sarcopenia guideline reported that the prevalence of sarcopenia was 4.1% to 11.5% in the general elderly population.^[2]^ Loss of lower leg muscle mass and grip strength are also risk factors for the development of VCF, and may be closely related to the pathogenesis of sarcopenia.^[3]^ The reported prevalence of sarcopenia in patients with VCF ranges from 33.9% to 65%.^[3–5]^

Handgrip strength (HGS) is associated with body musculature. Handgrip dynamometers are used in many studies for various purposes because they are easy to use and provide useful data. In oncology, HGS is associated with cancer-related fatigue,^[6]^ quality of life,^[7]^ postoperative complications,^[8]^ and mortality risk.^[8]^ We previously demonstrated that HGS is correlated with walking ability in lumbar spinal stenosis.^[9]^ Moreover, lower HGS may be associated with the onset of vertebral fracture.^[3]^ Women with distal radial fracture exhibit lower HGS and lower dynamic body balance capacity. In women with distal radial fracture who are at risk of future fragility fractures, HGS and poor dynamic body balance ability were found to be significant risk factors.^[10]^ A previous study demonstrated that HGS was the most important factor in the association between sarcopenia and osteoporosis, falls, and fractures.^[11]^ HGS in healthy elderly people was found to be greater than that in VCF patients.^[12]^ These results suggest that HGS may be a useful screening tool in VCF patients for assessing patient status and fracture risk.

Bioelectrical impedance analysis (BIA) is a useful, simple, and noninvasive tool for examining body composition. BIA is also used together with dual-energy X-ray absorptiometry to diagnose sarcopenia. Furthermore, BIA can be used to measure phase angle (PhA) and the distribution of body water between the extracellular water (ECW) and intracellular water (ICW) positions. Recent clinical studies have shown that PhA is a prognostic indicator in many clinical conditions (including nutritional risk, cancer, kidney disease, and human immunodeficiency virus infection) and in surgical patients.^[13–16]^ Segmental multi-frequency BIA can be used to precisely measure ICW and ECW and generate an “edema index.” This index is calculated as the ratio of extracellular water to total body water (ECW/TBW). A previous study demonstrated that ECW/TBW, as a combination of overhydration and protein-energy wasting, may be a significant predictor of poorer outcomes.^[17]^ However, the relationships between PhA and ECW/TBW in VCF patients are currently unclear.

A small number of studies have suggested that sarcopenia is associated with ADL in patients with VCF.^[18]^ However, no studies have reported a correlation between HGS and ADL in patients with VCF. The purpose of the current study was to investigate the relationship between HGS and ADL, balance, walking speed, calf circumference, body muscle, and body composition in patients with thoracolumbar VCF.

## 2. Methods

### 2.1. Patients

We retrospectively enrolled patients who were admitted at a single hospital for thoracolumbar VCF between October 2016 and March 2021. The study protocol was approved by the Ethics Review Board of Shiobara Spring Hospital. The present study was performed in accordance with the World Medical Association Declaration of Helsinki principles. We calculated the necessary sample size to achieve an alpha value of 0.05 and power of 0.80 using G*Power 3 statistical software (v 3.1.9.7, Heinrich-Heine-University, Düsseldorf, Germany),^[19,20]^ yielding an estimated sample size of 26. The inclusion criteria were as follows: patients with ≥ 1 recent symptomatic VCF (T5–L5); 60 years or older; and a low-energy injury (simple fall) or an injury without trauma. We defined osteoporotic VCF as axial compression of a vertebral body with an intact posterior restraining element including wedge, biconcave, and compression deformities as described by Eastell et al^[21]^ We assumed that the patient had acute VCF when they had tenderness at the fracture site without a callus on spinal radiography. The exclusion criteria were as follows: pathological fracture, including fractures related to malignancy, infection, or other medical conditions; burst fracture with a retro-pulsed bony fragment into the spinal canal; neurological deficit; use of steroids or medications for severe liver or kidney disease; no available BIA instrument; and no measures of fitness.

### 2.2. Measurements

HGS of both upper limbs were measured using a handheld dynamometer. The patients squeezed the dynamometer as hard as possible for 3 seconds. Two attempts were performed with each hand, with a brief rest between trials. We used the best performance for the analysis.^[22]^

Gait speed was measured using the timed 10-meter walk test. The 10-meter walk test measures the time that it takes a patient to walk 10 meters. We did not perform the tests in patients for whom it was difficult to perform them safely, such as those who needed walking aids, those who had recently had a lower limb fracture or surgery, or those who had neurological conditions. Trained physical therapists conducted the tests. For patients who could not perform the test, gait speed was recorded as 0 m/s. The Barthel Index is 1 of the most common rating scales for measuring activity limitations in patients with neuromuscular and musculoskeletal conditions. The Barthel Index measures 10 items of functioning in daily life, including feeding, bathing, grooming, dressing, toilet uses, transfers, mobility, and stair use. The Berg Balance Scale (BBS) was applied to evaluate standing and sitting balance, and each item was evaluated on a 5-point scale (a total of 56 points). Patients evaluated their average body pain using an 11-point numerical rating scale (0: no pain; 10: worst pain imaginable) on admission. Body mass index was calculated as weight/height^2^ (kg/m^2^). Calf circumference was measured at the thickest point on both sides. The mean of the right and left leg measurements was calculated.

### 2.3. Definition of sarcopenia

We used low HGS (< 28 kg for males and < 18 kg for females) and walking speed < 1.0 m/s to define sarcopenia in this study, according to the 2019 Asian Working Group for Sarcopenia.^[23]^ A multi-frequency validated BIA instrument, the Inbody S10 (Biospace, Seoul, Korea), was used to examine the patient in a supine position. This device was also used to measure skeletal muscle mass and fat mass. Skeletal muscle mass index (SMI) was the measured skeletal muscle mass divided by the square of the height in meters. Sarcopenia for men and women was defined as SMI values of < 7.0 kg/m^2^ and < 5.7 kg/m^2^, respectively. PhA, ECW, ICW, and TBW values were obtained using BIA. The ratio of ECW to TBW (ECW/TBW) and the ratio of ICW to TBW (ICW/TBW) were then calculated to compare the distribution of body water.

### 2.4. Statistical analysis

Continuous variables are presented as mean ±standard deviation. Correlations between HGS and the continuous variables were assessed using Pearson correlation coefficients. A *P* value of < 0.05 was considered to indicate statistical significance. All statistical analyses were performed using SPSS for Windows version 17.0 (SPSS, Chicago, IL).

## 3. Results

A total of 164 patients were initially assessed. We excluded 52 patients because of missing measures of fitness or because BIA was not available. We enrolled 112 patients (average age: 83.3 years; 26 males, 86 females). The total prevalence of sarcopenia was 61.6% (50% in male patients, 65.1% in female patients). We compared demographic parameters with HGS (Table 1). Female patients had significantly weaker HGS than male patients (*r* = −0.568, *P* < .001). In the cohort, there were significant correlations between HGS and age (*r* = −0.452, *P* < .001), height (*R* = 0.600, *P* < .001), weight (*R* = 0.258, *P* = .006), walking speed (*R* = 0.485, *P* < .001), and calf circumference (*R* = 0.491, *P* < .001). Fifty-nine patients were not able to walk 10 meters on admission. HGS was significantly correlated with Barthel Index (*R* = 0.430, *P* < .001) and BBS (*R* = 0.511, *P* < .001). HGS was not correlated with numerical rating scale of body pain. In male patients, HGS was significantly correlated with age (*r* = −0.585, *P* = .002), height (*R* = 0.486, *P* = .012), walking speed (*R* = 0.611, *P* = .001), calf circumference (*R* = 0.584, *P* = .003), Barthel Index (*R* = 0.582, *P* = .002), and BBS (*R* = 0.646, *P* = .005). In female patients, HGS was significantly correlated with age (*r* = −0.296, *P* = .006), height (*R* = 0.294, *P* = .006), weight (*R* = 0.248, *P* = .021), walking speed (*R* = 0.293, *P* = .006), calf circumference (*R* = 0.467, *P* < .001), Barthel Index (*R* = 0.338, *P* = .001), and BBS (*R* = 0.374, *P* = .004).

**Table 1:** Characteristics of Patients and Pearson Correlation Coefficients from Handgrip Strength.

	Total (n = 112)	*P*	*r*	Male (n = 26)	*P*	*r*	Female (n = 86)	*P*	*r*
Age (yr)	83.3 ± 7.8	<.001	−0.452	80.3 ± 9.2	.002	−0.585	84.3 ± 7.1	.006	−0.296
Gender (Male: Female)	26: 86	<.001	−0.568	-	-	-	-	-	-
Height (cm)	150.1 ± 8.7	<.001	0.600	161.2 ± 6.0	.012	0.486	146.8 ± 6.3	.006	0.294
Weight (kg)	50.7 ± 16.8	.006	0.258	60.4 ± 21.5	.678	−0.085	47.8 ± 14.0	.021	0.248
BMI (kg/m^2^)	22.4 ± 6.9	.607	0.049	23.4 ± 9.1	.359	−0.188	22.2 ± 6.2	.140	0.161
Grip (kg)	15.3 ± 7.3	-	-	22.8 ± 8.8	-	-	13.1 ± 4.9	-	-
Walking speed (m/s)	0.38 ± 0.48	<.001	0.485	0.59 ± 0.62	.001	0.611	0.31 ± 0.41	.006	0.293
Barthel index	58.3 ± 24.2	<.001	0.430	65.4 ± 26.1	.002	0.582	56.2 ± 23.3	.001	0.338
BBS	24.3 ± 18.0	<.001	0.511	31.8 ± 21.3	.005	0.646	22.1 ± 16.5	.004	0.374
NRS	6.0 ± 2.4	.346	−0.107	5.6 ± 2.7	.638	0.116	6.1 ± 2.3	.163	−0.181
Calf circumference (cm)	28.9 ± 3.9	<.001	0.491	30.1 ± 4.2	.003	0.584	28.6 ± 3.8	<.001	0.467

BBS = Berg Balance Scale, BMI = body mass index, NRS = Numerical Rating Scale, SD = standard deviation.

HGS was significantly correlated with the muscle mass of the right arm (*R* = 0.684, *P* < .001), left arm (*R* = 0.622, *P* < .001), trunk (*R* = 0.655, *P* < .001), right leg (*R* = 0.692, *P* < .001), and left leg (*R* = 0.673, *P* < .001) (Table 2). HGS exhibited a significant negative correlation with the ECW/TBW ratio (*r* = −0.498, *P* < .001). However, HGS was significantly positively correlated with the ICW/TBW ratio (*R* = 0.492, *P* < .001) and PhA (*R* = 0.550, *P* < .001). In male patients, HGS was significantly correlated with the muscle mass of the right arm (*R* = 0.467, *P* = .016), trunk (*R* = 0.391, *P* = .048), right leg (*R* = 0.625, *P* = .001), and left leg (*R* = 0.639, *P* < .001), and the ECW/TBW ratio (*r* = −0.457, *P* = .019), ICW/TBW ratio (*R* = 0.467, *P* = .016), and PhA (*R* = 0.653, *P* < .001). In female patients, HGS was significantly correlated with the muscle mass of the right arm (*R* = 0.544, *P* < .001), left arm (*R* = 0.497, *P* < .001), trunk (*R* = 0.507, *P* < .001), right leg (*R* = 0.429, *P* < .001), and left leg (*R* = 0.378, *P* < .001), and the ECW/TBW ratio (*r* = −0.419, *P* < .001), ICW/TBW ratio (*R* = 0.432, *P* < .001), and PhA (*R* = 0.426, *P* < .001).

**Table 2:** Pearson Correlation Coefficients between Handgrip Strength and Other Variables such as Body Muscle and Water Composition.

	Total (n = 112)	*P*	*r*	Male (n = 26)	*P*	*r*	Female (n = 86)	*P*	*r*
SMI (cm^2^/m^2^)	5.30 ± 1.15	<.001	0.629	6.39 ± 1.08	.003	0.556	4.97 ± 0.95	<.001	0.443
Muscle mas									
Right arm (kg)	1.5 ± 0.5	<.001	0.684	2.1 ± 0.4	.016	0.467	1.3 ± 0.4	<.001	0.544
Left arm (kg)	1.5 ± 0.6	<.001	0.622	2.1 ± 0.6	.123	0.311	1.3 ± 0.4	<.001	0.497
Trunk (kg)	14.3 ± 3.5	<.001	0.655	18.4 ± 3.0	.048	0.391	13.0 ± 2.5	<.001	0.507
Right leg (kg)	4.6 ± 1.4	<.001	0.692	6.3 ± 1.5	.001	0.625	4.1 ± 1.0	<.001	0.429
Left leg (kg)	4.6 ± 1.4	<.001	0.673	6.3 ± 1.4	<.001	0.639	4.1 ± 1.0	<.001	0.378
ICW (kg)	14.2 ± 3.5	<.001	0.569	18.1 ± 3.4	.096	0.333	13.1 ± 2.6	.001	0.349
ECW (kg)	9.8 ± 2.3	<.001	0.500	12.3 ± 2.3	.275	0.222	9.0 ± 1.7	.012	0.271
TBW (kg)	24.0 ± 5.8	<.001	0.543	30.4 ± 5.7	.151	0.290	22.1 ± 4.3	.003	0.319
ECW/TBW ratio	0.408 ± 0.009	<.001	−0.498	0.403 ± 0.010	.019	−0.457	0.410 ± 0.009	<.001	−0.419
ICW/TBW ratio	0.592 ± 0.009	<.001	0.492	0.596 ± 0.010	.016	0.467	0.591 ± 0.009	<.001	0.432
Phase angle (˚)	3.71 ± 0.83	<.001	0.550	4.15 ± 0.81	<.001	0.653	3.58 ± 0.79	<.001	0.426

ECW = extracellular body water, ICW = intracellular body water, SD = standard deviation, SMI = skeletal muscle index, TBW = total body water.

## 4. Discussion

In this study, we investigated the prevalence of sarcopenia in patients with VCF and examined the relationships between HGS and a range of factors. The findings revealed that among 112 enrolled patients, sarcopenia was present in 69 patients (61.6%). Moreover, HGS was correlated with sex, age, height, weight, walking speed, Barthel Index, BBS, calf circumference, SMI, muscle mass, ECW/TBW ratio, ICW/TBW ratio, and PhA.

Sarcopenia is a risk factor for osteoporotic VCF and has recently become a major issue in medical care for the elderly.^[24]^ The prevalence of sarcopenia ranges from 5.8% to 14.9% in men and from 4.1% to 16.6% in women, as reported using the relative appendicular skeletal muscle index or skeletal muscle index, according to the International Working Group on Sarcopenia or the European Working Group on Sarcopenia in Older People criteria.^[25]^ Thus, the prevalence of sarcopenia appears to vary depending on the diagnostic criteria used. Hida et al^[5]^ demonstrated that the prevalence rates of sarcopenia were 42% in 70-year-old patients with acute VCF and 25% in patients without acute VCF. The researchers reported that sarcopenia and lower leg muscle mass were risk factors for VCF.^[5]^ Eguchi et al^[3]^ found that decreased leg muscle mass and decreased HGS were risk factors for VCF in elderly women. Previous studies have reported a sarcopenia prevalence of 33.9% to 65% in VCF patients.^[3–5,26]^ In the present study, the prevalence was 61.6%, similar to that reported in previous studies.

The current results revealed that HGS was correlated with age, sex, height, weight, walking speed, and calf circumference in patients with VCF. Our previous study demonstrated that HGS was correlated with lower extension power, height, weight, and age in patients with lumbar spinal stenosis.^[9]^ HGS decreases considerably with age.^[27]^ Additionally, low muscle strength and power are strongly associated with 2 complementary definitions of poor athletic performance, regardless of age or sex. HGS is related to age, height, and weight, but not to body mass index. Moreover, positive correlations have been reported between calf circumference and HGS in geriatric patients (*R* = 0.422)^[28]^ and young Japanese women (*R* = 0.377).^[29]^

The present findings reveal that HGS is associated with the Barthel Index and BBS. Barthel Index is a scale used to measure ADL, and BBS is used to measure balance abilities. Thus, our findings suggest that HGS is associated with ADL and balance. In previous studies, HGS was significantly correlated with Barthel Index scores in frail elderly people (*R* = 0.214)^[30]^ and patients with dysphagia (*R* = 0.38).^[31]^ Moreover, HGS was significantly correlated with BBS in elderly people (*R* = 0.576).^[28]^ As a consequence, high HGS in elderly people is an indicator of good balance, and decreased HGS is an important risk factor for falls in postmenopausal women.^[32]^ These findings suggest that HGS is a good indicator of an individual’s ADL and balance.

In the current study, HGS was correlated with muscle mass measured by BIA. Our previous study revealed that HGS in patients with lumbar spinal stenosis was correlated with psoas muscle mass and skeletal muscle mass at the L3 level.^[9]^ Multiple studies have reported a close relationship between HGS and lifespan, whole-body muscle volume, and physical activity.^[33]^ The areas of the psoas and paraspinal muscles are important for grading the vitality of the patient. Low psoas muscle area is correlated with low HGS and short physical performance battery scores, indicating physical frailty.^[34]^ Low psoas muscle area is also related to prolonged hospital stays in elderly cardiac surgery patients. In frail patients classified on the basis of HGS, the total area of the psoas muscle was smaller than that of nonfrail patients.^[35]^

In the current study, HGS was related to PhA, ECW/TBW ratio, and ICW/TBW ratio in patients with VCF. Previously, the ECW/TBW ratio was reported to be significantly higher in patients with sepsis than healthy individuals, while PhA and the ICW/TBW ratio were significantly lower in patients with sepsis.^[36]^ In hepatic, pancreatic, and biliary surgery, an increased ECW/TBW ratio in patients with fluid imbalance suggests a possible causal relationship with the development of ascites and fluid retention in the postoperative period.^[37]^ A high ECW/TBW ratio was correlated with lower Subjective Global Assessment scores not only in patients receiving renal replacement therapy^[38]^ but also in patients with autosomal dominant polycystic kidney disease.^[39]^ Moreover, a high ECW/TBW ratio was associated with malnutrition according to the Subjective Global Assessment questionnaire. One study of peritoneal dialysis patients reported a cutoff value of ECW/TBW for 1-year mortality of > 0.371 for men and > 0.372 for women,^[40]^ and another study reported a cutoff value of 0.400.^[17]^ A study of acute heart failure patients revealed a cutoff value of ECW/TBW of 0.390^[41]^ for a higher incidence of rehospitalization. In this study, the mean ECW/TBW ratio was 0.408, suggesting that patients with VCF are in worse general condition. PhA was previously reported to be significantly and positively associated with somatic protein and muscle function in cancer patients.^[42]^ Another study reported that PhA was positively correlated with survival in patients undergoing hemodialysis.^[43]^ The ECW/TBW ratio and PhA have been used as indicators of poor systemic status and survival. To our knowledge, this is the first study to demonstrate the relationship between HGS, ECW/TBW ratio, and PhA.

We evaluated male and female patients across many variables in the current study. Many previous studies of VCF only evaluated female patients or grouped male and female patients together.^[3–5,12,18,44]^ HGS was more strongly correlated with walking speed, Barthel Index, BBS, ECW/TBW ratio, and PhA in male than female patients. This finding suggests that HGS is more critically related to ADL and body condition in male than female patients. Thus, male patients with VCF may be in worse condition and face more severe difficulties than female VCF patients.

The current study involved several limitations. First, patients were retrospectively surveyed at a single institution. Thus, the sample population may have been biased. Second, the cross-sectional design of the current study limited our ability to draw conclusions regarding causal relationships. In future, we plan to conduct a cohort study of VCF patients to enable the detection of causal factors. Third, the number of male patients with VCF in the current study was small. Including male VCF patients will be important in future studies of sarcopenia and frailty because they appear to have greater problems with sarcopenia and frailty than female VCF patients.

In conclusion, HGS was correlated with the Barthel Index in patients with thoracolumbar VCF. Thus, for thoracolumbar VCF patients, HGS provides an indicator of not only whole-body muscle strength but also ADL and balance. Moreover, PhA, ECW/TBW, and ICW/TBW were significantly correlated with HGS. Lastly, HGS was more strongly correlated with gait speed, Barthel Index, BBS, ECW/TBW ratio, and PhA in male than female patients.

## Acknowledgments

The authors thank Mr. Kabasawa and Mr. Sakaguchi, as well as the physical and occupational therapists at Shiobara Spring Hospital, for providing support in carrying out this study. We thank Benjamin Knight, MSc., and Carol Wilson, PhD, from Edanz (https://jp.edanz.com/ac) for editing a draft of this manuscript.

## Author contributions

**Conceptualization:** Hirokazu Inoue, Hideaki Sawamura, Yasuyuki Shiraishi, Ryo Sugawara, Atsushi Kimura, Katsushi Takeshita.

**Data curation:** Hirokazu Inoue, Yukinori Hayashi, Hideaki Sawamura.

**Formal analysis:** Hirokazu Inoue, Hideaki Watanabe.

**Supervision:** Masaaki Masubuchi, Katsushi Takeshita.

**Validation:** Hirokazu Inoue, Yukinori Hayashi, Hideaki Watanabe.

**Visualization:** Hirokazu Inoue, Yukinori Hayashi.

**Writing – original draft:** Hirokazu Inoue, Hideaki Watanabe.
